# Comparison of Handaxes from Bose Basin (China) and the Western Acheulean Indicates Convergence of Form, Not Cognitive Differences

**DOI:** 10.1371/journal.pone.0035804

**Published:** 2012-04-19

**Authors:** Wei Wang, Stephen J. Lycett, Noreen von Cramon-Taubadel, Jennie J. H. Jin, Christopher J. Bae

**Affiliations:** 1 Guangxi Museum of Nationalities, Nanning, China; 2 Department of Anthropology, School of Anthropology & Conservation, University of Kent, Canterbury, United Kingdom; 3 Joint POW/MIA Accounting Command, Central Identification Lab (JPAC-CIL), Hickam AFB, Hawai'i, United States of America; 4 Department of Anthropology, University of Hawai'i at Manoa, Honolulu, Hawai'i, United States of America; Illinois State University, United States of America

## Abstract

Alleged differences between Palaeolithic assemblages from eastern Asia and the west have been the focus of controversial discussion for over half a century, most famously in terms of the so-called ‘Movius Line’. Recent discussion has centered on issues of comparability between handaxes from eastern Asian and ‘Acheulean’ examples from western portions of the Old World. Here, we present a multivariate morphometric analysis in order to more fully document how Mid-Pleistocene (i.e. ∼803 Kyr) handaxes from Bose Basin, China compare to examples from the west, as well as with additional (Mode 1) cores from across the Old World. Results show that handaxes from both the western Old World and Bose are significantly different from the Mode 1 cores, suggesting a gross comparability with regard to functionally-related form. Results also demonstrate overlap between the ranges of shape variation in Acheulean handaxes and those from Bose, demonstrating that neither raw material nor cognitive factors were an *absolute* impediment to Bose hominins in making comparable handaxe forms to their hominin kin west of the Movius Line. However, the shapes of western handaxes are different from the Bose examples to a statistically significant degree. Moreover, the handaxe assemblages from the western Old World are all more similar to each other than any individual assemblage is to the Bose handaxes. Variation in handaxe form is also comparatively high for the Bose material, consistent with suggestions that they represent an emergent, convergent instance of handaxe technology authored by Pleistocene hominins with cognitive capacities directly comparable to those of ‘Acheulean’ hominins.

## Introduction

For over sixty years, reputed contrasts between the stone tools and artifacts made by Pleistocene hominins in eastern Asia versus the western Old World have inspired controversial discussion (e.g. [1], [2], [3], [4], [5], [6], [7], [8]). Work by Hallam Movius during the 1940s, formed the basis for much of this discussion [1], [9]. In particular, much attention was given to an alleged absence of “handaxes" in East and South East Asia. Handaxes are bifacially-worked and elongated implements, known from many Lower Palaeolithic sites in Africa, western Asia, and western Europe [10], [11]. Movius ([1]: 408 emphasis in original) underscored the widespread presence of handaxes in these regions in a particular manner, thus suggesting that “[i]t cannot be too strongly emphasized that it is the *absence* of certain characteristic types of implements" that formed sharp contrasts between the Palaeolithic records of eastern Asia when compared to those from the West. Moreover, Movius ([1]: 411) went on to state that the Palaeolithic records of East and South East Asia could be regarded as “monotonous and unimaginative", and even that these areas were “a region of cultural retardation". It has been argued that such phrases have influenced the nature of subsequent debate as much as archaeological considerations [8]. The ultimate naming of the so-called “Movius Line" by Carlton Coon ([12]: 48), appeared to reify the concept of a “cultural frontier" with putative biological, technological and cognitive distinctions between the hominin populations that lay on either side of it.

Since the time of Movius, however, there has been a growing awareness that elongated, bifacial artifacts are not entirely absent from eastern Asia (e.g. [3], [7], [13], [14], [15], [16], [17], [18]), although localities producing such examples still remain comparatively rare when considered against many regions west of the Movius Line [4], [19], [20]. In turn, discussion in recent years has increasingly refocused on issues of comparability (or lack thereof) between handaxes from eastern Asian and ‘Acheulean’ examples from the western Old World (e.g. [7], [8], [15], [21], [22], [23]).

It should be noted that the manufacture of specific stone artefacts in any given region can only ever provide an indication of the *minimum* cognitive capacities of their manufacturers (i.e. the production of relatively simple stone tools in any given context does not automatically imply that their manufacturers were cognitively diminished compared to Acheulean hominins). However, the manufacture of ‘Acheulean’ handaxes by Pleistocene fossil hominins is frequently suggested to imply the application of derived cognitive abilities (e.g. [24], [25]). Hence, robustly assessing the issue of comparability between handaxes from east of the Movius Line and ‘Acheulean’ examples might in turn shed light on issues of cognitive comparability between hominins across these geographic regions.

Handaxes from the Bose Basin (Guangxi Zhuang Autonomous Region, southern China) have played a prominent role in these recent debates (e.g. [15], [26]). Bose basin itself (23°33′–24°18′N, 106°7′–106°56′E), today cut through by the Youjiang River, is comprised of seven terraces containing fluvial, laterized deposits dating to the Late Pliocene and Pleistocene [15]. Stone artefacts, including handaxes, are limited exclusively to the middle and upper units of the fourth terrace [15], [27], [28]. Tektites securely dated by ^40^AR/^39^AR to 803,000±3000 years old are also exclusively limited to this fourth terrace [17]. This exclusive contextual association of tektites and handaxes makes the finds at Bose both the oldest and most securely dated examples of Pleistocene handaxe technology from East or Southeast Asia. In turn, this ensures that they are of paramount importance with regard to ongoing debates concerning the status and nature of handaxe technology east of the Movius Line.

Accurately determining the implications of handaxes from localities such as Bose for contemporary palaeoanthropological enquiry requires a robust comparative approach, whereby handaxes from different regions may be assessed directly in terms of overall affinity and comparability, and with statistical rigor. Here, we present a multivariate morphometric analysis in order to more fully document how the handaxes from the Bose Basin compare to handaxe examples from the west, as well as additional (Mode 1) cores from across the Old World. A total of 468 artifacts were subjected to a Procrustes-based geometric morphometric analysis using a configuration of 51 semilandmarks. Two sets of statistical analyses were undertaken. In the first instance, Principal Components Analysis (PCA) was used to assess the overall extent and pattern of morphological variability across specimens. In a second analysis, a cluster phenogram was used to further compare degrees of relative morphological similarity and difference between artifacts.

## Results

**Figure 1:**
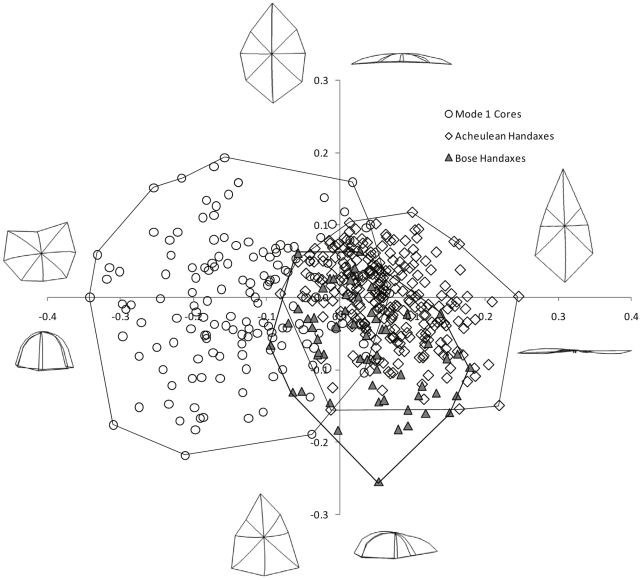
Results of principal components analysis (PC1 = *x*-axis, PC2 = *y*-axis). Shape variability of western Acheulean handaxes, Bose handaxes, and Mode 1 cores on PC1 and PC2 (31.4% and 13.4% of variance explained respectively). Wireframes indicate artifact shape variations associated with the extremities of each PC. Polygons illustrate maximum extent of shape variability for each artifact class.

In the first analysis, the shapes of handaxes from the western Acheulean localities and Mode 1 cores from the Old World localities were subjected to PCA along with 56 handaxes from Bose. Figure 1 shows the results of the PCA analysis for PCs 1 and 2. As Figure 1 shows, the major patterns of distinction between the artifacts as a whole is between the Mode 1 cores falling toward one end of PC1 and the handaxe artifacts (both the western examples and the Bose specimens) falling toward the opposite end of PC1, with handaxes (from whatever region) being generally more elongated and less domed than the Mode 1 cores. Importantly, PC scores for both the Acheulean handaxes and the Bose handaxes are significantly different from those of the Mode 1 cores on PC1 (Table 1). Figure 1 also demonstrates that there is evident overlap in the shape of Bose handaxes compared with western Acheulean examples. However, PC scores for the Bose handaxes and the Acheulean specimens are significantly different on PC2 (Table 1).

**Table 1:** Mann-Whitney U test comparisons (p-values in parentheses) of differences between Mode 1 cores (*n* = 157), Acheulean (*n* = 255) and Bose (*n* = 56) handaxes on the first two Principal Components.

	Principal Component 1
Mode 1 cores vs. Acheulean Handaxes	1403.5 (exact p<0.0001)
Mode 1 cores vs. Bose Handaxes	620.5 (exact p<0.0001)

	Principal Component 2
Acheulean vs. Bose Handaxes	2677.5 (exact p<0.0001)

It is also evident from Figure 1, that on a comparative basis, the handaxes from Bose are more variable as a group than the handaxes from the west. This is further supported via examination of the standard deviations (Table S1) of PC scores compared across the first 22 PCs (accounting for 95% of total shape variation), which demonstrates that with only one exception (PC14) the handaxes from Bose are consistently more variable across all PCs than the Acheulean handaxes, taken as a group. F-tests found that Mode 1 cores were statistically more variable (p<0.0001) than the Acheulean and Bose handaxes combined for each of the 22 principal components tested, while the Bose handaxes were statistically more variable (p<0.01) than the Acheulean handaxes for 16 of the 22 principal components tested.

**Figure 2:**
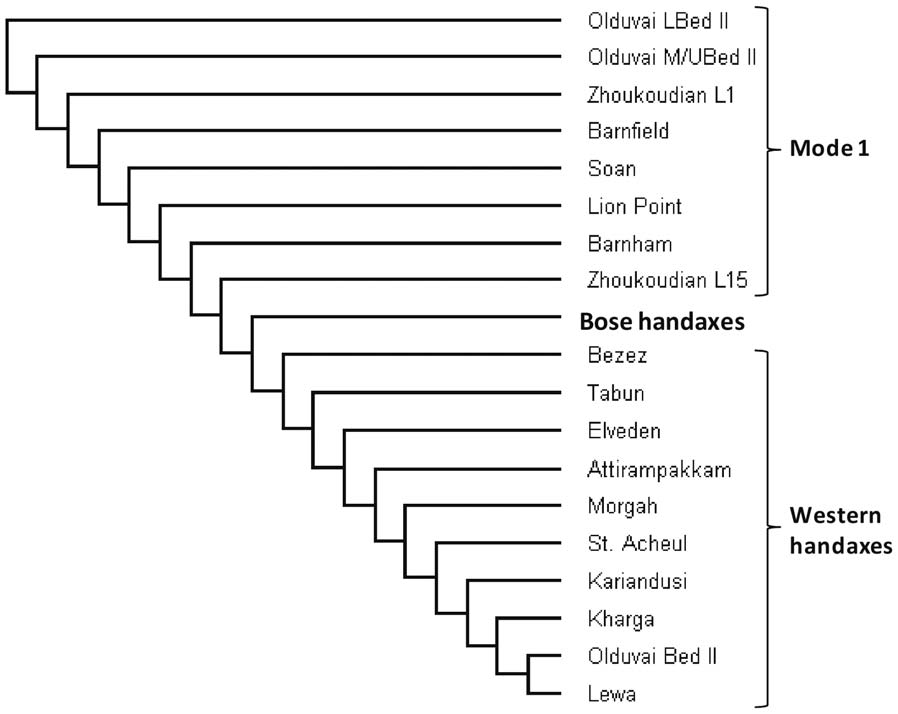
Neighbor-joining cluster diagram based on Mahalanobis distances.

In a second analysis, a neighbor-joining [29] phenogram was generated based on Mahalanobis distances between the assemblages (Figure 2). This analysis demonstrates that each of the handaxe assemblages from the western Old World are all more similar to one another than any individual assemblage is to the Bose handaxes. In other words, the Bose material is not more similar to any particular western handaxe assemblage to a greater extent than the western handaxes are, on average, to each other.

## Discussion

The results of our analyses demonstrate several crucial points regarding the behavioral and cognitive abilities of the Pleistocene hominins of the Bose Basin. They also shed light on factors regarding the appearance of handaxes at this geographic locality ∼803 thousand years ago.

The first point of note is that in the PCA analysis both the western handaxes and the Bose handaxes are clearly situated toward one end of the main axis of variation (PC1), and that both sets of handaxes are significantly different from the Mode 1 cores in this respect. Moreover, there is a gross comparability and overlap in terms of shape between the Bose handaxes and the western handaxes as a whole. There is substantial contextual, experimental, use-wear, and morphological evidence that artifacts classified as ‘handaxes’ were used as functional cutting/chopping tools during the Pleistocene [11], [30], [31], [32], [33], [34], [35], [36], [37], [38], [39]. This does not negate the possibility that handaxe production also provided a source of utilizable flakes ([40]:196), but there is clear evidence that the form of artifacts comprising the main diagnostic element of the western ‘Acheulean’ (i.e. handaxes) was driven by deliberate, functional considerations pertinent to their demonstrated role as tools in cutting/chopping activities. The gross comparability of form (or *Bauplan*) evident in our analyses between western handaxes and those from Bose, thus suggests that the general shape of the Bose handaxes was also the product of functional considerations relating to chopping and/or cutting activities.

The evident overlap in the shape of Bose handaxes and the western handaxes unequivocally demonstrates two further points. Raw material factors have long been considered to influence the form of stone artifacts [41], and issues relating to raw material have also been considered as putative causes for the Movius Line (for review see [4], [8]). Our results, however, support other recent research (e.g. [42]) suggesting that raw material factors did not provide an absolute constraint in eastern Asia to the production of handaxe forms directly comparable to those found west of the Movius Line. In addition, this evident overlap of handaxe form demonstrates that strictures of hominin cognition do not provide a convincing case for factors relating to the Movius Line. In sum, neither raw material nor cognitive factors were an *absolute* impediment to Bose hominins in making comparable handaxe forms to their hominin kin west of the Movius Line.

Our analyses also provide insight into the probable factors underlying the appearance of handaxes at Bose. Despite considerable overlap in the shapes of handaxes from Bose and those from the west, overall patterns of shape variability between the two sets of handaxes were, however, found to be significantly (p<0.0001) different. Moreover, shape variability in the Bose handaxes is greater overall than the variability exhibited collectively by the western Acheulean handaxes. It is also notable that our second analysis demonstrated that the handaxes from the different western Acheulean localities were, on average, all more similar to each other than any of them were to the Bose handaxes. The presence of handaxes at sites in eastern Asia such as Bose has been attributed to two distinct mechanisms: dispersal east by Acheulean hominins versus independent technological convergence [15], [20], [22], [23]. High levels of morphological variability in the earliest occurrences of specific stone tool traditions have been observed in other contexts, whereby it is noted that initial periods of experimentation with a particular form leads to higher levels of variability when compared with subsequent manifestations of the same tradition [43]. Only in subsequent phases do functional considerations lead to a relative stabilization of form and a reduction in overall variability following this initial period of relatively greater morphological disparity. Given the extended timeframe of the Acheulean [44], [45], similar general processes could have been in operation (even if somewhat weaker than is being observed in lithic artifacts from later contexts), accounting for the lower variation observed in the Acheulean data relative to the Bose material. The patterns observed in our data are, therefore, more consistent with the Bose handaxes representing an emergent and convergent instance of handaxe technology, than they are with the dispersal of Acheulean hominins into southern China.

In sum, our analyses demonstrate that in terms of shape variability, the Mid-Pleistocene handaxes from Bose display patterns of both evident similarity and dissimilarity compared with western Acheulean examples. In our comparative analyses, there is sufficient overlap in shape variability between the Bose handaxes and western Acheulean to suggest a gross comparability in terms of functionally-related form. Moreover, this shape overlap demonstrates that neither raw material differences, nor factors relating to hominin cognition, can be seen as unconditional constraints on the production of handaxes in eastern Asia, directly comparable in shape to those seen in western contexts. However, the relatively high levels of shape variability evident in the Bose handaxes are consistent with them being an emergent instance of technological convergence with the western Acheulean. Hence, at least in the case of the Bose material, the appearance of handaxe technology east of the Movius Line should not be used to support a scenario involving the dispersal by hominins from the west into East Asia, but rather seen as an independent invention authored by Pleistocene hominins with cognitive capacities directly comparable to those of western Acheulean hominins. Such conclusions are particularly important given increased recognition (e.g. [26], [46], [47], [48]) of the essential value of the eastern Asian record for furthering our understanding of human evolution and hominin interaction during the Pleistocene.

## Materials and Methods

### Materials

56 handaxes from Bose Basin were used in the analyses and were compared against handaxes from western Acheulean localities in Africa, Europe, western Asia and the Indian subcontinent, as well as Mode 1 cores (Table 2). It should be emphasized that the aim of our analyses was not to assess variation *within* the western Acheulean assemblages; such analysis has already been undertaken for this material in a series of previous papers, which have included consideration of variation patterns, raw material patterns (or lack thereof), etc. in these data (see e.g. [21], [49], [50]). Rather, the primary aim of our analyses was specifically to use the Acheulean artifacts in a comparative manner in order to assess the relative comparability and variation of the Bose material against this specific empirical backdrop.

**Table 2:** Comparative samples used in the analyses alongside the 56 handaxes from Bose Basin, China.

Locality	*n*	Raw material	Artifact class
Barnfield Pit, Kent, UK	22	Chert	Mode 1 cores
Barnham St. Gregory, Suffolk, UK	30	Chert	Mode 1 cores
Lion Point, Clacton, Essex, UK	18	Chert	Mode 1 cores
Olduvai Gorge (Lower Bed II), Tanzania	11	Lava, chert, quartz	Mode 1 cores
Olduvai Gorge (Middle/Upper Bed II), Tanzania	26	Lava, chert, quartz	Mode 1 cores
Soan Valley, Pakistan	25	Quartzite	Mode 1 cores
Zhoukoudian, Locality 1, China	14	Sandstone, quartz, limestone	Mode 1 cores
Zhoukoudian, Locality 15, China	11	Sandstone, quartz	Mode 1 cores
Attirampakkam, India	30	Quartzite	Handaxes
Bezez Cave (Level C), Adlun, Lebanon	30	Chert	Handaxes
Elveden, Suffolk, UK	24	Chert	Handaxes
Kariandusi, Kenya	30	Lava	Handaxes
Kharga Oasis (KOl0c), Egypt	17	Chert	Handaxes
Lewa, Kenya	30	Lava	Handaxes
Olduvai Gorge (Bed II), Tanzania	13	Quartz, lava	Handaxes
Morgah, Pakistan	21	Quartzite	Handaxes
St. Acheul, France	30	Chert	Handaxes
Tabun Cave (Ed)	30	Chert	Handaxes

The Bose handaxes (*n* = 35 quartzite, 21 sandstone) were recovered from three localities; Fengshudao, Damei and Lucidao [17], [27]. The two handaxes from Damei were excavated examples and found in direct stratigraphic association with tektites [17]. A further five handaxes from Fengshudao were also excavated, while all other specimens from this locality and Lucidao were surface collected. The average mass of the Bose handaxes examined is 1174.43 grams (range = 479–2120 grams; standard deviation = 383.4 grams). This material is curated by the Guangxi Natural History Museum, Nanning. Over the course of the past quarter century, more than 100 unifacially and bifacially worked so-called ‘Large Cutting Tools’ (LCTs) have been surface collected and excavated from various sites within the Bose Basin, many of which have been referred to as ‘handaxes’ by different researchers. However, in order to be consistent across the different geographic regions (including the Bose Basin), we considered particular specimens as ‘handaxes’ if they conformed to established (although broad) archaeological definitions for such artifacts (e.g. [10], [33], [37], [51], [52], [53]). That is, artifacts were classified as ‘handaxes’ when they were found to be ‘tear-drop’, ‘triangular’ or ‘ovate’ in planform, and lenticular or triangular in cross-section, as well as exhibiting a bifacial series of flake removals resulting in the imposition of the long axis of the piece. Artifacts were classified as ‘Mode 1 cores’ [54] when several flakes (≥3) had been removed from a lithic mass/nuclei (such as a cobble or nodule) yet did not conform to the definition of handaxes, nor could be identified as another category of core artifact (e.g. Levallois core) typically excluded from the extremely broad Mode 1 range of artifacts; this definition is thus broad enough to include ‘chopper’ and ‘polyhedron’ style cores [55].

### Geometric morphometrics

The basis of geometric morphometrics is the identification and quantification of ‘homologous landmarks’, defined as “a point of correspondence on an object that matches between and within populations" ([56]: 3, [57]). 51 geometrically-defined 3D co-ordinates (semilandmarks) were recorded using a Crossbeam Co-ordinate Caliper [58]. The resulting landmark configuration is shown in Figure S1. Full details regarding the semilandmarking protocol, orientation of artefacts, and definitions of all landmarks can be found in Refs. [58], [59].

Landmark configurations were subjected to generalized Procrustes analysis (GPA) and tangent space projection in Morphologika 2.5 (http://life.biosunysb.edu/morph/soft-3d.html; [60]). GPA proceeds by removing variation between landmark configurations due to isometric scale by reducing all configurations to unit centroid size, and then implements least-squares criteria to minimize residual differences between configurations due to translation and rotation [61], [62]. Any remaining variation between homologous landmark positions (Procrustes residuals) is then regarded as shape variation between configurations.

### Analyses

Two analyses were undertaken. In the first analysis, Procrustes residuals were subjected to principal components analysis (PCA) in Morphologika 2.5 based on the total covariance matrix. PCA enables the major shape variation between individual objects (in this case lithic nuclei) to be examined in a hierarchical fashion, whereby the first PC describes the major axis of shape variation (size having already been controlled for), the second PC describes the second major axis of variation, and so on. PCA was therefore employed here in a comparative sense in order to examine overall patterns of shape variability and affinity between the major artefactual classes, and the Bose specimens.

In the second analysis, Mahalanobis distances [63] between the assemblages were computed and a neighbor-joining phenogram [29] was generated in PHYLIP 3.66 (J. Felsenstein, http://evolution.genetics.washington.edu/phylip) based on these distances. The purpose of this analysis was to determine if the Acheulean handaxes were all more similar to each other than any individual set was to the Bose material. This thus allowed a comparative analysis of affinity within the various ‘handaxe’ sets, complementary to that undertaken in the PCA.

## Supporting Information

Figure S1 **Configuration of 51 landmarks used in the 3D geometric morphometric analyses.**
(TIF)

Table S1 **Morphometric variability (standard deviations) of each of three major groups of stone tools compared across each of the first 22 Principal Components (accounting for 95% of the total morphometric variation).** Mode 1 cores consistently showed the greatest variability across all PCs compared with Acheulean and Bose handaxes. F-tests found that for each Principal Component Mode 1 variability was significantly greater (p<0.0001) than the variability of the Acheulean handaxes and the Bose handaxes combined. Moreover, in the case of all but one PC (PC 14) the handaxes from Bose were also consistently more variable across all PCs than the Acheulean handaxes. F-tests further found that Bose handaxes were statistically more variable (p≤0.01) than the Acheulean handaxes for all principal components except for PCs 1–4, 14 and 16.
(DOCX)
