# Supplementary figures and images for: Comparison of Handaxes from Bose Basin (China) and the Western Acheulean Indicates Convergence of Form, Not Cognitive Differences
Source: PLoS One. 2012 Apr 19;7(4):e35804. doi: 10.1371/journal.pone.0035804 (PMC3334908; doi:10.1371/journal.pone.0035804)

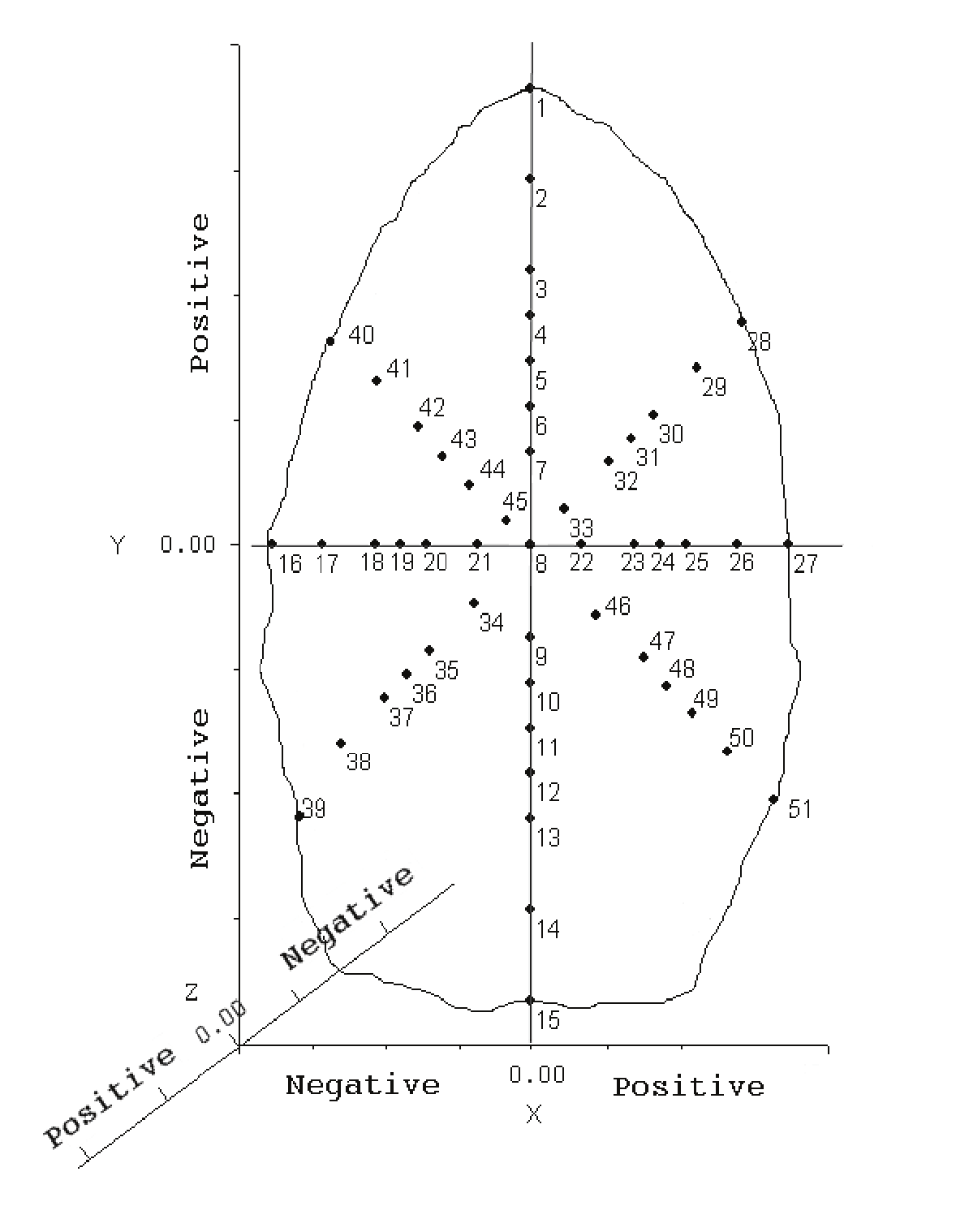

Supplement: Figure S1 — Configuration of 51 landmarks used in the 3D geometric morphometric analyses. (TIF) [file pone.0035804.s001.tif]
