# Supplementary material for: Comparison of Handaxes from Bose Basin (China) and the Western Acheulean Indicates Convergence of Form, Not Cognitive Differences
Source: PLoS One. 2012 Apr 19;7(4):e35804. doi: 10.1371/journal.pone.0035804 (PMC3334908; doi:10.1371/journal.pone.0035804)
Supplement: Table S1 — Morphometric variability (standard deviations) of each of three major groups of stone tools compared across each of the first 22 Principal Components (accounting for 95% of the total morphometric variation). Mode 1 cores consistently showed the greatest variability across all PCs compared with Acheulean and Bose handaxes. F-tests found that for each Principal Component Mode 1 variability was significantly greater (p<0.0001) than the variability of the Acheulean handaxes and the Bose handaxes combined. Moreover, in the case of all but one PC (PC 14) the handaxes from Bose were also consistently more variable across all PCs than the Acheulean handaxes. F-tests further found that Bose handaxes were statistically more variable (p≤0.01) than the Acheulean handaxes for all principal components except for PCs 1–4, 14 and 16. (DOCX) [file pone.0035804.s002.docx]

**Table S1**

**Table S1.** Morphometric variability (standard deviations) of each of three major groups of stone tools compared across each of the first 22 Principal Components (accounting for 95% of the total morphometric variation). Mode 1 cores consistently showed the greatest variability across all PCs compared with Acheulean and Bose handaxes. F-tests found that for each Principal Component Mode 1 variability was significantly greater (p<0.0001) than the variability of the Acheulean handaxes and the Bose handaxes combined. Moreover, in the case of all but one PC (PC 14) the handaxes from Bose were also consistently more variable across all PCs than the Acheulean handaxes. F-tests further found that Bose handaxes were statistically more variable (p≤0.01) than the Acheulean handaxes for all principal components except for PCs 1-4, 14 and 16.

| Principal Component | Mode 1 Cores | Acheulean Handaxes | Bose Handaxes | % variance explained by each PC |
| --- | --- | --- | --- | --- |
| **PC 1** | 0.095 | 0.060 | 0.063 | 31.41 |
| **PC 2** | 0.086 | 0.060 | 0.071 | 13.38 |
| **PC 3** | 0.068 | 0.060 | 0.062 | 9.25 |
| **PC 4** | 0.084 | 0.036 | 0.040 | 7.73 |
| **PC 5** | 0.071 | 0.028 | 0.035 | 5.42 |
| **PC 6** | 0.065 | 0.025 | 0.029 | 4.29 |
| **PC 7** | 0.053 | 0.023 | 0.035 | 3.62 |
| **PC 8** | 0.049 | 0.024 | 0.030 | 3.09 |
| **PC 9** | 0.050 | 0.020 | 0.026 | 2.72 |
| **PC 10** | 0.040 | 0.018 | 0.022 | 1.93 |
| **PC 11** | 0.036 | 0.020 | 0.024 | 1.75 |
| **PC 12** | 0.040 | 0.016 | 0.020 | 1.69 |
| **PC 13** | 0.036 | 0.016 | 0.019 | 1.56 |
| **PC 14** | 0.034 | 0.016 | 0.015 | 1.30 |
| **PC 15** | 0.030 | 0.015 | 0.018 | 1.17 |
| **PC 16** | 0.027 | 0.015 | 0.016 | 1.02 |
| **PC 17** | 0.028 | 0.011 | 0.014 | 0.85 |
| **PC 18** | 0.028 | 0.010 | 0.012 | 0.76 |
| **PC 19** | 0.027 | 0.009 | 0.011 | 0.73 |
| **PC 20** | 0.024 | 0.010 | 0.012 | 0.64 |
| **PC 21** | 0.024 | 0.009 | 0.010 | 0.59 |
| **PC 22** | 0.022 | 0.010 | 0.011 | 0.53 |
